# Morning and Evening Blue-Enriched Light Exposure Alters Metabolic Function in Normal Weight Adults

**DOI:** 10.1371/journal.pone.0155601

**Published:** 2016-05-18

**Authors:** Ivy N. Cheung, Phyllis C. Zee, Dov Shalman, Roneil G. Malkani, Joseph Kang, Kathryn J. Reid

**Affiliations:** 1 Department of Neurology, Northwestern University, Chicago, Illinois, United States of America; 2 School of Medicine, Case Western Reserve University, Cleveland, Ohio, United States of America; 3 Department of Preventive Medicine-Biostatistics, Northwestern University, Chicago, Illinois, United States of America; Pennsylvania State University, UNITED STATES

## Abstract

Increasing evidence points to associations between light-dark exposure patterns, feeding behavior, and metabolism. This study aimed to determine the acute effects of 3 hours of morning versus evening blue-enriched light exposure compared to dim light on hunger, metabolic function, and physiological arousal. Nineteen healthy adults completed this 4-day inpatient protocol under dim light conditions (<20lux). Participants were randomized to 3 hours of blue-enriched light exposure on Day 3 starting either 0.5 hours after wake (n = 9; morning group) or 10.5 hours after wake (n = 10; evening group). All participants remained in dim light on Day 2 to serve as their baseline. Subjective hunger and sleepiness scales were collected hourly. Blood was sampled at 30-minute intervals for 4 hours in association with the light exposure period for glucose, insulin, cortisol, leptin, and ghrelin. Homeostatic model assessment of insulin resistance (HOMA-IR) and area under the curve (AUC) for insulin, glucose, HOMA-IR and cortisol were calculated. Comparisons relative to baseline were done using t-tests and repeated measures ANOVAs. In both the morning and evening groups, insulin total area, HOMA-IR, and HOMA-IR AUC were increased and subjective sleepiness was reduced with blue-enriched light compared to dim light. The evening group, but not the morning group, had significantly higher glucose peak value during blue-enriched light exposure compared to dim light. There were no other significant differences between the morning or the evening groups in response to blue-enriched light exposure. Blue-enriched light exposure acutely alters glucose metabolism and sleepiness, however the mechanisms behind this relationship and its impacts on hunger and appetite regulation remain unclear. These results provide further support for a role of environmental light exposure in the regulation of metabolism.

## Introduction

The widespread use of electric lighting and our modern 24/7 lifestyle is accompanied by self-imposed changes to patterns of light-dark exposure (e.g. limited daytime exposure to sunlight and increased nocturnal light exposure) [1–3], which are thought to play a role in health [4, 5]. The impacts of light exposure on hunger and metabolism are of particular interest. There are at least two potential mechanisms by which alterations in light-dark exposure may impact hunger and metabolism, one is via the circadian system, and the second is via physiological arousal.

The circadian system controls the timing of physiological and behavioral rhythms such as rest and activity patterns [6], alertness [7], hunger and appetite [8], and glucose and insulin sensitivity [9, 10]. Perturbations to these rhythms have been associated with poor health [11–15]. The strongest exogenous modulator of the central circadian clock is the pattern of light-dark exposure [16, 17], with the circadian system most sensitive to blue-wavelength light [18–20]. Not only does light-dark exposure change across the day, but the wavelength of light also varies across the day [21].

Light exposure also impacts the level of physiological arousal, such as sleepiness, alertness [7, 22], and cortisol levels [23, 24]. Cortisol levels also influence hepatic and peripheral tissue insulin sensitivity and, consequently, circulating glucose levels [25, 26], thereby providing another possible path by which light exposure may impact metabolism.

Recent studies in humans have examined the impact of light exposure on body weight and metabolism. Studies manipulating morning light exposure are typically associated with leaner body weight, lower body fat and altered appetite outcomes [27–29]. On the other hand, later timing of light tends to be associated with heavier body weight. Recent work from our group in adults reports an association between the timing of habitual light exposure and body mass index (BMI); people with a later mean timing of light exposure over 500 lux (MLiT500) had a higher BMI [30]. Furthermore, shift work, which is often accompanied by increased light exposure at night, is associated with higher BMI [31] and risk of obesity [32]. However, few studies have examined the impact of evening light, as opposed to light at night, on hunger, weight regulation, and metabolism. There is significant variation in hunger across the day, corresponding with endogenous circadian rhythms; specifically, a 17% difference in hunger between the morning trough and evening peak [8]. As such, the impact of light exposure on hunger should be studied at different times of day.

Animal studies suggest that the timing of light exposure impacts the timing of food intake and therefore metabolism, which in turn influences weight regulation [33, 34]. For example, mice subjected to constant light show an increase in food intake during the normal rest period, decreased energy expenditure, loss of circadian variation in insulin sensitivity, and higher body weight gain, despite similar overall caloric intake compared to mice kept in a normal light-dark cycle. However, weight and fat gain even while under constant light were attenuated when food intake was restricted to the normal active period [33].

Although evidence from both human and animal studies suggests that light exposure modulates food intake, metabolic function, and weight regulation, most studies have examined light exposure at only one time of day despite known circadian variations in these functions. Additionally, these studies examine impacts of light exposure over time, leading us to postulate that acute actions of light exposure (that may lead to these longer term outcomes) may occur via immediate alterations to hunger and/or metabolism. Therefore, the aim of this study was to compare the acute effects of 3 hours of morning versus evening blue-enriched light exposure on hunger, metabolic function, and physiological arousal. We hypothesized that morning blue-enriched light would decrease overall hunger and positively impact metabolic function, while evening blue-enriched light would increase hunger and negatively impact metabolic function and that these effects would be associated with changes in physiological arousal, namely cortisol and sleepiness levels.

## Methods

The Northwestern University Institutional Review Board approved this study and all participants provided written informed consent.

### Participants

Nineteen healthy adults were recruited from the Chicago area with fliers and newspaper advertisements. Initial eligibility was determined via telephone screening. Participants were then interviewed during a screening visit to determine their medical, psychiatric, social, and medication history. Final eligibility was determined by the study physician.

In order to qualify for the study, participants were required to be healthy and between 18–50 years of age and to have regular eating and sleeping schedules. Regular eating pattern was defined as: 1) three main meals per day, at approximately the same time every day; 2) two or fewer snacks per day; and, 3) two or fewer snacks after 8pm per week. Regular sleep schedule was defined as: 1) rest start between 9pm-1am; 2) rest end between 5am-9am; 3) rest duration between 6.5–8.5 hours; and, 4) no more than one nap per week.

Exclusion criteria were: 1) any sleep, cognitive, neurological, or major psychiatric disorder; 2) significant depressive symptoms (Beck Depression Index-II [35] (BDI-II) score > 20); 3) endocrine dysfunction, gastrointestinal disease, blindness or significant vision impairment; 4) shift work; 5) current use within the past month of psychoactive, hypnotic, stimulant or analgesic medications; 6) obesity (BMI > 30 kg/m^2^); 7) current use of light therapy; 8) sleep apnea (apnea hypopnea index (AHI) ≥ 15); 9) periodic leg movements (movement arousal index ≥ 15); 10) any unstable or serious medical conditions; 11) a history of habitual smoking (6 or more cigarettes per week) or drinking (7 or more alcoholic beverages per week) or caffeine consumption of greater than 300 mg per day; and 12) use of any other legal or illicit substance that may affect sleep and/or appetite. Due to the metabolic stress associated with pregnancy and breastfeeding, patients who were pregnant or breastfeeding were also excluded.

Participants’ sleeping and eating schedules were monitored for seven days using actigraphy (Actiwatch-L and Spectrum, Philips Respironics), sleep diaries, and food logs. Actiwatches were set with 30-sec epoch length and medium sensitivity to determine average weekday rest start time, rest end time, and total sleep time. Participants were asked to record free day or work day status, wake up time, whether or not an alarm was used, naps, bed time, sleep time, times awake during the night, and a rating of ease of falling asleep and waking up in a daily sleep diary. Food logs were used to determine food intake and timing. Participants were asked to record the time of each meal, the meal type (i.e. breakfast, lunch, dinner, or snack), location, food type, brand, and amount including water and other beverages.

Maintenance of habitual sleeping and eating schedule was confirmed by actigraphy, sleep diaries, and food logs for an additional seven days immediately prior to the Clinical Research Unit (CRU) admission.

### Experimental Protocol

#### General Protocol

**Fig 1:**
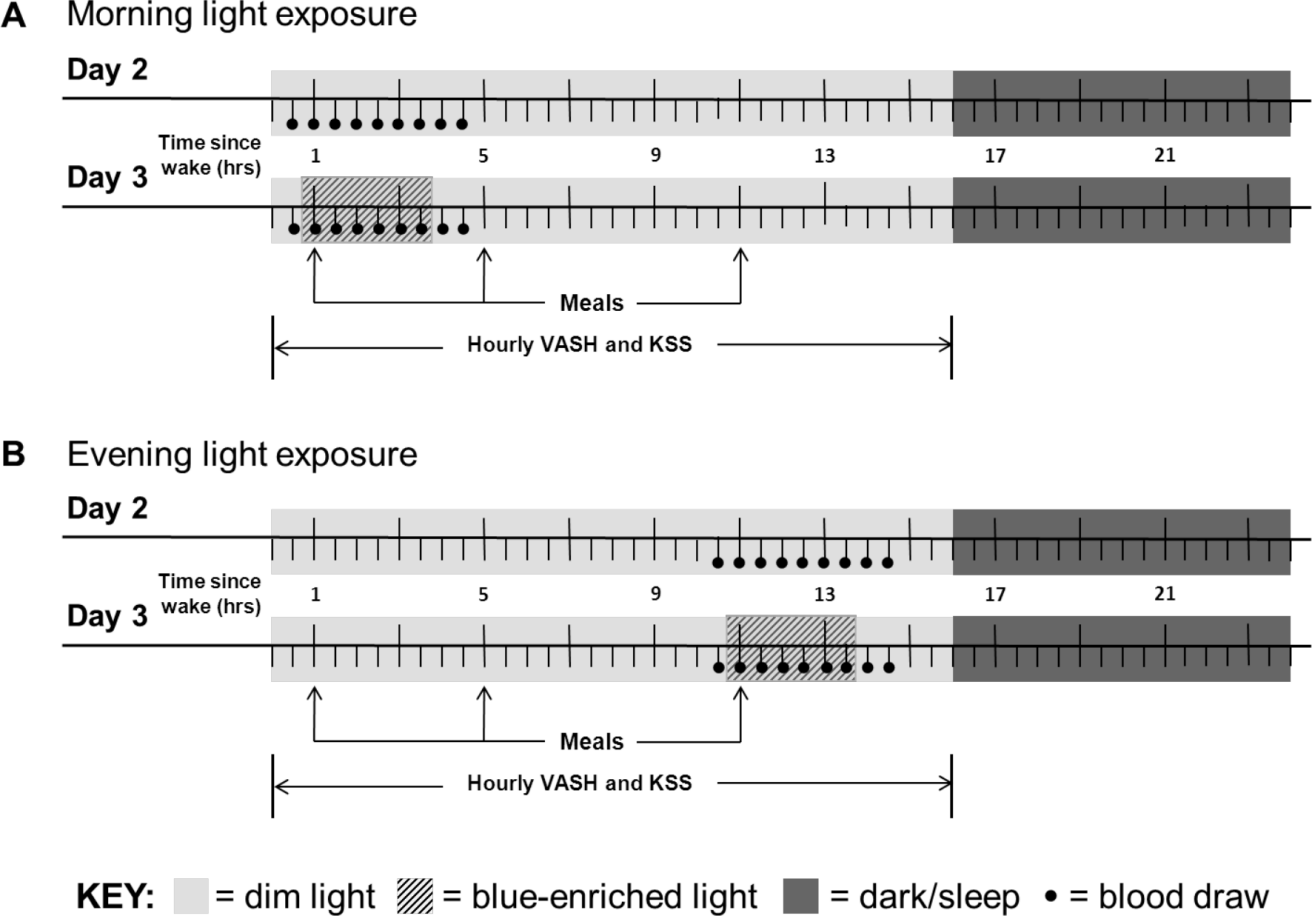
**Schematic representation of the experimental protocol for morning (A) and evening (B) light exposure.** Participants arrived in the early evening of Day 1 (not shown) and began dim light < 20 lux (light grey shading) on Day 2 during 16 hours of wake and < 3 lux (dark grey shading) during 8 hours of sleep. Fixed isocaloric meals were given 1, 5, and 11 hours after wake. On both Days 2 and 3, questionnaires (VASH and KSS) were collected hourly during wake and blood was drawn (black circles) at 30-minute intervals 0.5–4.5 hours after wake for morning light exposure participants (A) and 10.5–14.5 hours after wake for evening light exposure participants (B). Participants were exposed to 3 hours of blue-enriched light (hashed shading on Day 3) starting 0.5 hours after wake for morning light exposure participants (A) and starting 10.5 hours after wake for evening light exposure participants (B) compared to dim light (Day 2). Participants were discharged on Day 4 (not shown). Abbreviations: VASH–visual analogue scale for hunger, KSS–Karolinska Sleepiness Scale.

Participants who met eligibility criteria were randomized to either the morning or evening group for this initial study. Participants completed a 4-day, 3-night stay in the Northwestern Memorial Hospital CRU in Chicago, Illinois (Fig 1) from March 23, 2012 to March 22, 2013. Participants were admitted to the CRU at approximately 18:00 on Day 1 and bed time was determined from their average weekday rest onset time recorded from actigraphy in the week immediately prior to their CRU visit. Participants received fixed isocaloric meals 1, 5, and 11 hours after wake with fixed macronutrient composition of 50–55% carbohydrate, 15–20% protein, and 30–35% fat. Meals were prepared by the CRU research bio-nutritionist based on height, weight, usual caloric intake, and estimated energy expenditure from the Harris-Benedict equation [36]. While the content of breakfast, lunch, and dinner meals were different, the content of a specific meal was the same on each experimental day (i.e. breakfast on Days 2 and 3 was identical, but was different from lunch). Participants consumed at least 90% of their meals and there were no statistically significant differences in overall and carbohydrate intake on Days 2 and 3. No snacking or caffeine was allowed during the study. Water and sugar-free, caffeine-free sodas were provided ad libitum.

All participants had dim light on Day 2 and then blue-enriched light during the scheduled light exposure period (morning or evening) on Day 3. On Day 2, starting 8 hours after their sleep onset, participants began a schedule of 16 hours of dim light (<20 lux) during wake and 8 hours of dark (<3 lux) during sleep. On Day 3, participants were also exposed to blue-enriched light for three hours beginning 0.5 hours after wake (morning group) or 10.5 hours after wake (evening group). Visual analogue scale for hunger (VASH) and the Karolinska Sleepiness Scale (KSS) were given hourly, starting upon waking. On Days 2 and 3, blood was sampled for measurement of glucose, insulin, cortisol, leptin, and ghrelin every 30 minutes for four hours (in association with the light exposure period) for a total of 9 samples. This sampling interval allowed for a fasting blood sample, a blood sample with light exposure before the meal, several blood samples following the meal during light exposure, and two samples after the light exposure. The first blood sample taken each day was immediately prior to the start of the scheduled light exposure window and was considered the baseline for that day. Blood samples were processed and frozen at -80°C until assay. Participants were discharged from the CRU on the morning of Day 4.

#### Light Exposure

All participants were kept in dim light during wake for the duration of their stay in the CRU, except for blue-enriched light exposure on Day 3 that was achieved with two goLITE BLU Plus light boxes (Philips, Amsterdam, Netherlands) and normal overhead room lighting (260 lux). The goLITE BLU Plus is a 5.5 x 5.5 inch device containing 60 blue light emitting diodes (LEDs) with a peak wavelength of 468 ± 8nm and a half-peak bandwidth of 20nm. Participants were seated upright facing a table. Light boxes were positioned on the table top 24 inches to the left and right peripheral side of the seated participant. Measurement of 24 inches distance from the light box was done at a downward angle from eye level to the light box. Light exposure was conducted as follows: overhead lights were turned on and the light from the light boxes was gradually increased using the four intensity settings on the light boxes in 5-minute increments at 280, 320, 340, and 370 lux and similarly tapered down in a reversed manner starting 15 minutes prior to completion of the three-hour light exposure condition. Participants were monitored closely by research staff during light exposure conditions and were instructed to face forward and keep eyes open while engaging in seated activities such as reading or watching movies. Light exposure in lux at the eye level was monitored using a lux meter (Minolta Auto Light Meter IV F) during dim light condition set ups and throughout the blue-enriched light exposure period.

### Outcome Measures

#### Screening and Demographics

Wrist activity monitoring was conducted for one week of screening and one additional week immediately prior to the CRU visit using actiwatches (Actiwatch-L and Spectrum, Philips Respironics) to measure average weekday rest start time, rest end time, and total sleep time. Actigraphy analysis was done using Actiware (version 5, Philips Respironics) with the aid of the sleep log, and a valid day was defined as one without any off-wrist time during the rest interval and with less than 4 hours of off-wrist time in a 24-hour period. Midpoint of sleep was calculated as the average time between rest start time and rest end time. Time above light threshold (TALT) of 1,000 lux for white light was calculated as the average minutes per day with light exposure above 1,000 lux for the week of recording.

Height and weight were taken by clinical research staff upon arrival to the CRU and body mass index was calculated as [weight (kg)/(height (m))^2^].

#### Hunger

The VASH [37] assesses participants’ hunger, desire to eat, amount of food that they could eat, and fullness. A hunger composite score (Hcomp) was calculated using the formula Hcomp = [(hunger) + (desire) + (amount) + 100 –(full)]/4 to yield an Hcomp score between 0–100, with a higher value indicative of greater hunger. The VASH was given hourly.

Leptin was measured with a Millipore human leptin radioimmunoassay (RIA) kit, with standards from 0.5–100 ng/mL, inter-assay precision of 3.5–6.2%, and intra-assay precision of 3.4–8.4%. Ghrelin was measured with a Millipore total ghrelin RIA kit, with standards from 150 to 9600 pg/mL, inter-assay precision of 14.7–17.8%, and intra-assay precision of 3.3–10.0%.

#### Metabolic Function

Glucose was measured using the glucose oxidase method on a Beckman CX3D Analyzer. Insulin was measured by chemiluminescent immunoassay on a Siemens Immulite 2000 with sensitivity limited to 14.4 pmol/L. Blood samples were taken for 4 hours at 30 minute intervals. The first sample was taken 30 minutes before the scheduled light exposure period and the second sample was taken 30 minutes later during light exposure but before the meal. To determine the impact of light following a meal, a meal was given 30 minutes after the first sample. The third to seventh samples were taken during the light exposure period and the eighth to ninth samples were taken following the light exposure period.

The homeostatic model of assessment for insulin resistance (HOMA-IR) was calculated as glucose (mg/dl) x insulin (μU/mL)/405, using the second sample on each day immediately prior to the meal, which was after 30 minutes of blue-enriched light exposure on Day 3.

When used for diagnostic purposes, HOMA-IR is validated under fasting conditions. However, experimentally HOMA-IR has been used to compare relative insulin resistance under the following conditions: post breakfast, midday meal and evening meal in sleep restricted versus rested states [38], as HOMA-IR total area across 24 hours in sleep restricted versus rested states [39], and to measure insulin resistance 0–6 hours after the breakfast meal in patients with type 2 diabetes [40].

For the morning group, the HOMA-IR is fasting (14 hour fast between the evening meal at 11hrs after wake and the following morning’s meal at 1hr after wake), while for the evening group HOMA-IR is calculated following a 5.5 hour fast. In a previous study, a reasonable correlation between glucose, insulin, and HOMA-IR values from overnight fast samples and samples taken 4 hours after the lunch meal [41] have been shown. Given the findings of this previous study, the glucose, insulin, and HOMA-IR values from the evening group are relevant for relative comparison within and between the groups.

#### Measures of Arousal

Sleepiness level was assessed hourly using the Karolinska Sleepiness Scale (KSS) [42]. Participants rated their level of sleepiness on a scale from 1 (extremely alert) to 9 (very sleepy, great effort to keep awake). The KSS is a widely used scale that closely correlates with physiological measures of sleepiness [43].

Cortisol was measured by chemiluminescent immunoassay on a Siemens Immulite 2000 with sensitivity limited to 1 ug/dL.

### Data Analysis and Calculations

To account for individual variability, VASH composite scores, leptin, ghrelin, glucose, insulin, and cortisol are shown as a change from baseline within each light condition (dim or blue-enriched light). For each measure, baseline was the sample immediately prior to the specified light exposure. All other variables (VASH component scores, HOMA-IR, area under the curve outputs, KSS, etc.) were analyzed as raw values.

Area under the curve (AUC) analysis was conducted using Prism (GraphPad, version 4.0) across samples 2–9 for glucose, insulin, and cortisol, and for samples 3–9 for HOMA-IR (to exclude the fasting samples and include only glucose and insulin values following meal ingestion) as a measure of the HOMA-IR response to meals [38]. Variables used for analysis include total area, peak value, and peak time for glucose and insulin, and total area for HOMA-IR and cortisol during the morning and evening dim and blue-enriched light exposure conditions.

Statistical analyses were done with descriptive statistics, t-tests, Chi-squared tests, Pearson correlations, and repeated measures ANOVAs using SPSS (SPSS Inc., version 16). Statistical significance was defined as p<0.05 using two-tailed tests.

Descriptive statistics were conducted for participant demographics and characteristics from the screening week and the week prior to the CRU visit, and differences were analyzed with two-sample t-tests for continuous variables and with Chi-squared tests for categorical variables.

Analysis of variables related to the intervention (excluding AUC variables) were conducted on values calculated as a change relative to baseline. Baseline was considered to be the first sample before the start of the light exposure period.

Differences between dim and blue-enriched light conditions were analyzed with paired t-tests for all variables except for the differences in the VASH hunger composite and component scores, which were assessed with repeated measures ANOVA (time x day). Correlations of the change between blue-enriched and dim light conditions were examined with Pearson correlations (sleepiness or cortisol with hunger or HOMA-IR).

Differences between the morning and evening groups were assessed as a change from baseline using two-sample t-tests at two time points: 1) during light exposure but prior to the meal (which was 15 minutes (questionnaires) or 30 minutes (blood samples) into light exposure), and 2) approximately 2.5 hours into the light exposure period (which was approximately 2 hours post meal). These time points were selected to allow for a fasting baseline blood sample, a blood sample with light exposure before the meal, and blood samples following the meal during light exposure. The Karolinska Sleepiness Scale (KSS) was missing for one morning and one evening group participant. A single blood sample was missing for one morning group participant and out of range insulin levels for one participant in the morning group were removed. One morning group participant had an Actiwatch failure during the week prior to the CRU stay. For each analysis, the number of subjects is indicated in the tables.

## Results

### Participant Demographics and Characteristics

Participant characteristics are listed in Table 1. Nineteen healthy adult participants (20–39 years of age with median age of 28 years) completed the study. For the morning group (n = 9), there were 5 females, and the mean age was 26.0 ± 4.4 years with a mean BMI of 24.2 ± 2.8 kg/m^2^. For the evening group (n = 10), there were 6 females, and the mean age was 29.9 ± 6.1 years with a mean BMI of 23.8 ± 3.2 kg/m^2^. Average weekday rest start time, average weekday rest end time, average weekday midpoint of sleep, and average weekday total sleep time for the morning and evening groups were 23:40 ± 0:36 and 23:32 ± 0:49, 7:22 ± 0:53 and 7:15 ± 0:56, 3:31 ± 0:38 and 3:23 ± 0:50, and 403.8 ± 41.8 minutes and 409.7 ± 45.0 minutes, respectively. Time above light threshold (TALT) of 1,000 lux for white light for the morning and evening groups were 46.54 ± 35.21 minutes and 32.44 ± 33.87 minutes, respectively. There were no significant differences between the morning and evening groups in terms of demographics and other characteristics.

**Table 1:** Participant demographics and characteristics shown as M±SD or No. (%).

	Morning (n = 9)	Evening (n = 10)	p-value^b^
Participant Demographics			
Age (years)	26.0 ± 4.4	29.9 ± 6.1	0.13
Body mass index (kg/m^2^)	24.2 ± 2.8	23.8 ± 3.2	0.79
Gender (n(%))			
Female	5 (56%)	6 (60%)	0.68
Male	4 (44%)	4 (40%)	
Race (n(%))			
American Indian/Alaskan Native	0 (0%)	0 (0%)	0.43
Asian	2 (22%)	2 (20%)	
Native Hawaiian or Pacific Islander	0 (0%)	0 (0%)	
Black or African American	1 (11%)	0 (0%)	
White	6 (67%)	8 (80%)	
Actigraphic Characteristics^a^			
Rest start time (hh:mm)	23:40 ± 0:36	23:32 ± 0:49	0.67
Rest end time (hh:mm)	7:22 ± 0:53	7:15 ± 0:56	0.81
Midpoint of sleep	3:31 ± 0:38	3:23 ± 0:50	0.72
Total sleep time (min)	403.8 ± 41.8	409.7 ± 45.0	0.77
Time above white light of 1,000 lux (min)^c^	46.54 ± 35.21	32.44 ± 33.87	0.40

a. Weekday averages from one week of actigraphy during screening.

b. P-values shown are from two-sample t-tests for continuous variables (age, body mass index, rest start time, rest end time, midpoint of sleep, total sleep time, and time above white light of 1,000 lux) and from Chi-squared tests for categorical variables (gender and race).

c. N = 9 for time above white light of 1,000 lux in the evening group.

### Hunger

**Fig 2:**
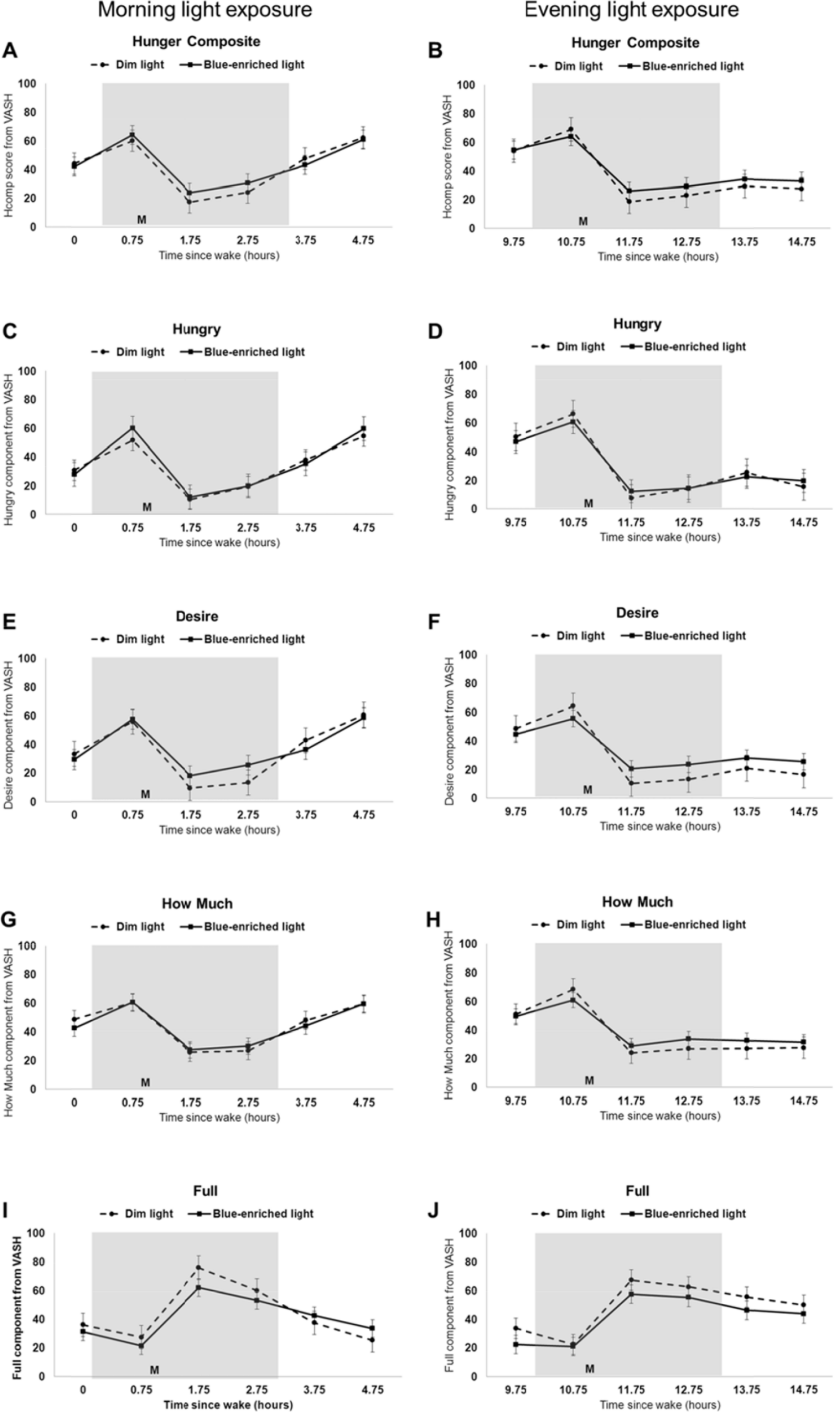
Hunger in dim versus blue-enriched morning and evening light. Subjective hunger composite scores (A-B) and component scores (C-J) during dim light (dotted line) compared to blue-enriched light (solid line) for morning (left panels; n = 9) and evening (right panels; n = 10) participants. Grey shading indicates specified light exposure condition; M denotes meal given 1 or 11 hours after wake for morning and evening participants, respectively. Results are plotted as mean ± standard error.

Subjective hunger was not significantly different between blue-enriched light exposure and dim light exposure conditions or between morning and evening groups (Fig 2A and 2B). Repeated measures analysis of variance (ANOVA) for the hunger composite scores and the component scores (hungry, desire, how much, and full; Fig 2C–2J) indicated a time effect but neither a day effect nor a time by day effect.

### Leptin and Ghrelin

There were no significant differences in leptin or ghrelin levels between blue-enriched light exposure and dim light exposure conditions or between morning and evening groups (Table 2).

**Table 2:** Change from baseline (prior to start of light exposure) in leptin and ghrelin measures, between conditions (dim versus blue-enriched light) within each group and between groups (morning versus evening).

	Group								
	Morning (n = 9)			Evening (n = 10)					
	Condition						Between groups comparison		
	Dim	Blue-enriched	p-value	Dim	Blue-enriched	p-value	Morning^c^	Evening^c^	p-value
Leptin (ng/mL)									
30min Light^a^	-2.97 ± 8.54	-1.68 ± 2.40	0.58	-0.34 ± 0.98	-0.38 ± 1.56	0.96	1.29 ± 6.79	-0.03 ± 2.05	0.56
2.5hrs Light^b^	-0.46 ± 1.81	-1.66 ± 1.96	0.26	2.43 ± 2.96	1.64 ± 3.94	0.45	-1.19 ± 2.97	-0.79 ± 3.13	0.78
Ghrelin (pg/mL)									
30min Light^a^	-51.4 ± 172.9	46.7 ± 198.8	0.40	-43.4 ± 108.1	39.9 ± 148.9	0.15	98.11 ± 333.9	83.3 ± 168.0	0.90
2.5hrs Light^b^	-147.1 ± 222.8	-116.3 ± 186.4	0.64	-167.5 ± 155.8	-117.2 ± 139.5	0.35	30.8 ± 192.0	50.3 ± 160.3	0.81

All values are shown as mean ± standard deviation as a change from baseline.

a. 30min Light refers to change relative to baseline from the time point approximately 30 minutes after the onset of the specified light exposure condition.

b. 2.5hrs Light refers to change relative to baseline from the time point approximately 2 hours post meal and 2.5 hours into the 3 hour specified light exposure condition.

c. Difference between blue-enriched and dim light conditions relative to baseline.

### Metabolic Function

**Fig 3:**
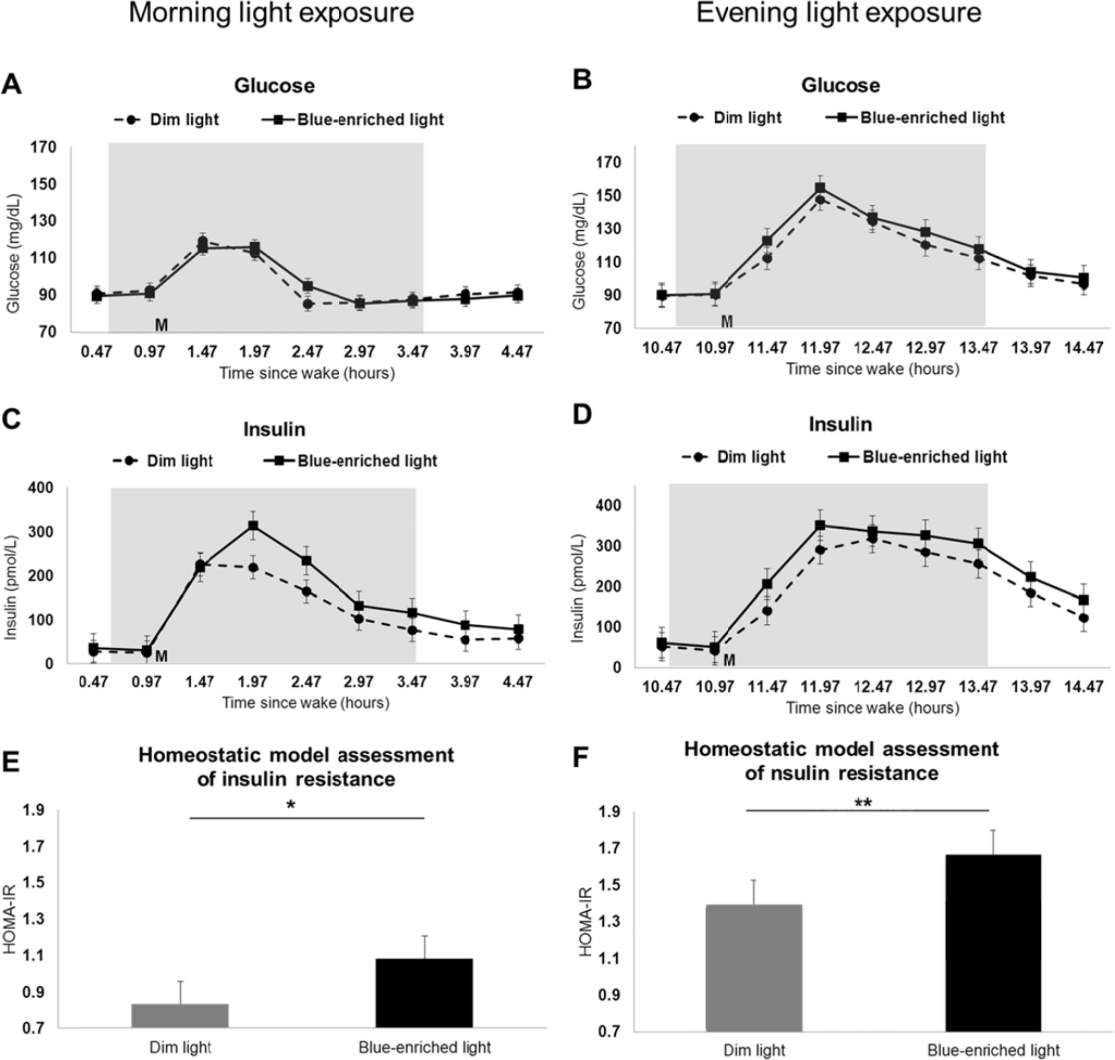
Glucose, insulin, and HOMA-IR in dim versus blue-enriched morning and evening light. Glucose levels (A-B), insulin levels (C-D), and homeostatic model assessment of insulin resistance (E-F) during dim light (dotted line on line plots and grey bar on bar graph) compared to blue-enriched light (solid line on line plots and black bar on bar graph) for morning (n = 9; n = 8 for insulin and HOMA-IR) and evening (n = 10) participants. Grey shading on line plots indicates specified light exposure condition; M on line plots denote meal given 1 or 11 hours after wake for morning and evening participants, respectively. Results are plotted as mean ± standard error. *p<0.05, **p<0.01.

Mean (± standard error) glucose and insulin levels for each sample of the blue-enriched and dim light conditions are shown in Fig 3A–3D. Homeostatic model assessment of insulin resistance (HOMA-IR) values during the blue-enriched and dim light conditions are shown in Fig 3E and 3F.

#### Morning group

AUC analysis indicated no differences in glucose total area, peak value, or peak time (Table 3) in the blue-enriched light compared to dim light condition. The insulin total area was larger (35,127 versus 25,914 pmol*min/L, p<0.01; Table 3) but there were no differences in insulin peak value or peak time with blue-enriched light exposure compared to dim light condition.

**Table 3:** Glucose, insulin, HOMA-IR, and cortisol between conditions (dim versus blue-enriched light) within each group and between groups (morning versus evening).

	Group								
	Morning (n = 9)			Evening (n = 10)					
	Condition						Between groups comparison		
	Dim	Blue-enriched	p-value	Dim	Blue-enriched	p-value	Morning^f^	Evening^f^	p-value
Glucose									
Total area^a^ (mg*min/dL)	20,280 ± 2184	20,321 ± 1,441	0.92	24,630 ± 3,35160	25,776 ± 4,666	0.27	41.7 ± 1,269	1,146 ± 3,114	0.34
Peak value (mg/dL)	128 ± 13	122 ± 7.5	0.14	148 ± 24	162 ± 33	0.02	-6.11 ± 11.0	13.8 ± 15.6	<0.01
Peak time (hrs since wake)	1.69 ± 0.26	1.69 ± 0.26	1.00	12.1 ± 0.2	12.0 ± 0.3	0.68	0 ± 0.43	-0.1 ± 0.37	0.79
Insulin^a^									
Total area^b^ (pmol*min/L)	25,914 ± 12,151	35,127 ± 16,347	<0.01	46,833 ± 22,494	55,885 ± 25,529	0.02	9,213 ± 7,279	9,052 ± 10,333	0.97
Peak value (pmol/L)	271 ± 130	325 ± 130	0.42	385 ± 193	449 ± 227	0.18	53.7 ± 71.4	64.8 ± 140	0.84
Peak time (hrs since wake)	2.00 ± 0.71	2.00 ± 0.25	1.00	12.3 ± 0.5	12.4 ± 0.7	0.61	0 ± 0.75	0.1 ± 0.61	0.75
HOMA-IR^a^^c^									
30min Light^d^	0.83 ± 0.56	1.08 ± 0.47	0.04	1.40 ± 0.91	1.66 ± 0.96	<0.01	0.25 ± 0.27	0.27 ± 0.19	0.87
Total area^e^	994 ± 538	1307 ± 619	0.03	2145 ± 1291	2741 ± 1716	0.04	313 ± 338	595 ± 771	0.35
Cortisol (mg/dL)									
Total area^b^	2292± 711	2178 ± 408	0.58	1206± 741	972 ± 438	0.28	-114 ± 591	-234 ± 645	0.68

Values are shown as mean ± standard deviation.

a. N = 8 for insulin and HOMA-IR measures in the morning group

b. Total area refers to the area under the curve calculated across eight half-hourly blood samples from 1–4.5 hours after wake (for the morning group) or 11 to 14.5 hours after wake (for the evening group) during the specified light exposure condition.

c. Homeostatic model of assessment for insulin resistance (HOMA-IR) was calculated for dim and blue-enriched light exposure conditions as [glucose (mg/dl) x insulin (μU/mL)/405].

d. 30min Light time point refers to HOMA-IR calculated with values for glucose and insulin coming from the blood sample taken approximately 30 minutes after the onset of the specified light exposure condition and immediately prior to the morning meal given 1 hour after wake for the morning group or immediately prior to the evening meal given 11 hours after wake for the evening group.

e. Total area for HOMA-IR refers to the area under the curve calculated across seven half-hourly blood samples from 1.5–4.5 hours after wake (for the morning group) or 11.5 to 14.5 hours after wake (for the evening group) during the specified light exposure condition.

f. Difference between blue-enriched and dim light conditions.

At 30 minutes after light onset and just prior to the meal, HOMA-IR increased more from baseline with blue-enriched light exposure compared to dim light condition (1.08 versus 0.83, p = 0.04; Table 3). Also, HOMA-IR total area was higher with blue-enriched light exposure compared to dim light condition for the morning group (1307 versus 994, p = 0.03; Table 3).

#### Evening group

Glucose peak value was higher (162 versus 148 mg/dL, p = 0.02; Table 3) but there was no difference in glucose total area or peak time with blue-enriched light exposure compared to dim light condition. Insulin total area was larger (55,885 versus 46,833 pmol*min/L, p = 0.02; Table 3), but there was no difference in insulin peak value or peak time with blue-enriched light exposure compared to dim light condition.

At 30 minutes after light onset and just prior to the meal, HOMA-IR increased more from baseline with blue-enriched light exposure compared to dim light condition (1.66 versus 1.40, p<0.01; Table 3). Also, HOMA-IR total area was higher with blue-enriched light exposure compared to dim light condition for the evening group (2741 versus 2145, p = 0.04; Table 3).

#### Between Groups

Between groups comparison in response to blue-enriched light relative to dim light indicated that the evening group had significantly higher glucose peak value than the morning group (13.8 versus -6.11 mg/dL, p<0.01; Table 3). There were no other significant differences between the morning and evening groups for glucose or insulin when comparing blue-enriched and dim light conditions (Table 3).

### Physiological Arousal

#### Sleepiness

**Fig 4:**
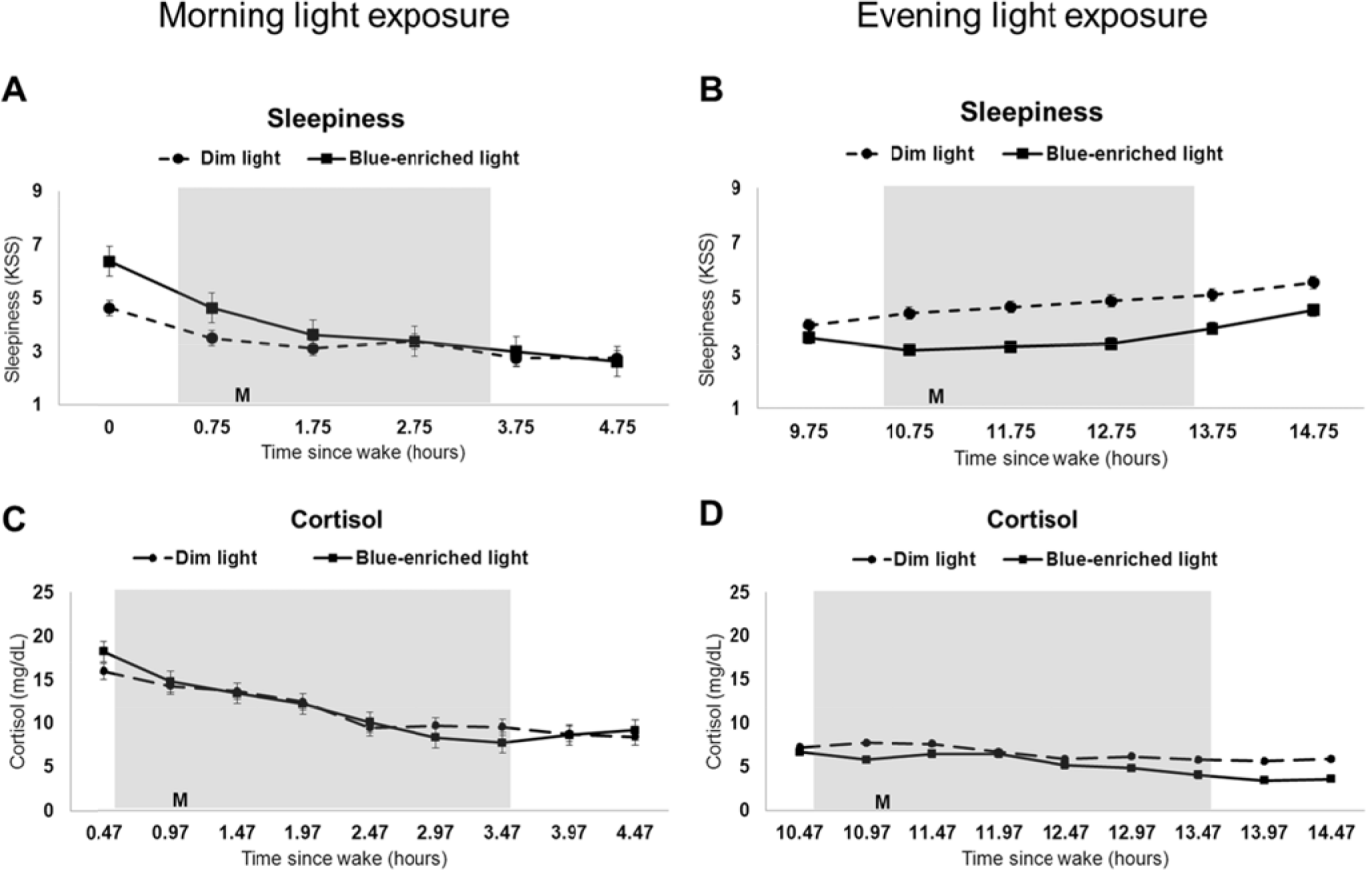
Sleepiness and cortisol in dim versus blue-enriched morning and evening light. Subjective sleepiness (A-B) and cortisol levels (C-D) during dim light (dotted line) compared to blue-enriched light (solid line) for participants in the morning (n = 8 for sleepiness and n = 9 for cortisol) and evening (n = 9 for sleepiness and n = 10 for cortisol) groups. Grey shading indicates specified light exposure condition; M denotes meal given 1 or 11 hours after wake for morning and evening participants, respectively. Results are plotted as mean ± standard error.

Subjective sleepiness was reduced more with the blue-enriched light exposure compared to the dim light condition in both the morning and evening groups (Fig 4A and 4B).

In the morning group, sleepiness was reduced more with blue-enriched light exposure than with dim light exposure (Fig 4A), however sleepiness started higher at baseline for the blue-enriched light compared to the dim light condition.

In the evening group, sleepiness was decreased 15 minutes after onset of blue-enriched light exposure and remained lower for one hour after lights returned to dim conditions (all p<0.05) compared to dim light exposure (Fig 4B).

#### Cortisol

There were no significant differences in cortisol levels between blue-enriched light exposure and dim light exposure conditions or between morning and evening groups (Fig 4C and 4D and Table 3).

### Associations between sleepiness and cortisol with hunger or HOMA-IR

There were no significant correlations between the change in the measures of arousal (sleepiness or cortisol) from the blue-enriched light and dim light condition with hunger or HOMA-IR.

## Discussion

Blue-enriched light exposure acutely altered metabolic function in both the morning and the evening compared to dim light. Specifically, both the morning and evening blue-enriched light exposure resulted in higher insulin resistance relative to dim light. In the evening group, blue-enriched light also led to higher peak glucose compared to dim light, suggesting an inability of insulin to adequately compensate for the increase in glucose at this time of day.

While blue-enriched light clearly altered subjective alertness in the evening, the lack of effect on subjective hunger was surprising considering the findings from a previous study showing changes in subjective appetite with morning bright light exposure [27]. It is possible that, unlike subjective alertness, a single acute light exposure may not be sufficient to illicit changes in subjective hunger. However, repeated daily exposures or exposure while in a natural environment (as opposed to a laboratory setting with controlled food intake and little physical activity) may lead to different outcomes more in line with these prior field-based studies.

There was also no effect of blue-enriched light exposure on hormonal measures of hunger/satiety (leptin and ghrelin). This is in contrast to findings from Figueiro and colleagues [28] reporting changes in leptin and ghrelin in response to morning red, green, and blue light following sleep restriction (5 hours of time in bed). This difference could be explained by the sleep restriction in their study, while the participants in the current study were given more opportunity to sleep (8 hours of time in bed) prior to light exposure.

In both rodents [11, 33] and humans [30], daily light exposure patterns are associated with body weight, body composition, and metabolism even after taking caloric intake into account. Given these reports, light’s influence on weight regulation likely involves other mechanisms beyond a potential increase in caloric intake due to changes in hunger/appetite. One possible mechanism is the impact of blue-enriched light exposure on glucose metabolism, as observed in these earlier animal studies and in the current study. The metabolic response to blue-enriched light exposure was quick; within 30 minutes of blue-enriched light exposure onset, HOMA-IR was 30% higher in the morning and 19% higher in the evening compared to dim light exposure. It is plausible that extended or more chronic exposure to blue-enriched light may further impact insulin resistance, given that HOMA-IR is 50–65% higher in the morning with repeated sleep restriction compared to the fully rested state [38, 44]. These changes in insulin resistance with blue-enriched light exposure could, over time, impact feeding behaviors, body composition, and/or body weight.

The differential effect of morning compared to evening light on peak glucose and the subsequent possibility that participants are less able to maintain glucose with increased insulin secretion in the evening may be due to the fact that glucose levels are naturally greater following the evening meal than the morning meal [9], which influences the ability of insulin to bring down glucose levels during evening light exposure. While the effects of a single pulse of blue-enriched light in the morning or the evening on glucose and insulin levels suggest that light exposure later in the day may be detrimental [30], they do not necessarily support the idea of morning light being beneficial [27, 29]. It is also unclear whether these effects would continue to be observed with repeated daily exposures.

The lack of change in cortisol in response to blue-enriched light exposure was surprising given our hypothesis that the effects of light exposure on hunger and metabolism may occur via increases in sympathetic activation, in particular via light exposure’s effects on cortisol. There are, however, several potential explanations for why we observed no significant change. One possibility is that we may not have sampled cortisol frequently enough. If cortisol drove the effects on glucose metabolism that were observed within 30 minutes of light exposure, cortisol would need to change almost immediately following onset of light exposure, and 30 minute sampling of cortisol may not be sufficient. Another explanation is that the effects of light exposure may not have a significant effect on cortisol; evidence of this in the literature is equivocal, with some studies showing decreases in cortisol [23, 45] while others show increases in cortisol [24]. Finally, Fonken and colleagues concluded that the reductions in corticosterone that they observed in mice under conditions of bright light for 8 weeks were due to masking of the glucocorticoid rhythm by food intake and that changes in glucocorticoid concentrations are probably not necessary for altered metabolism [33].

Despite the lack of change in cortisol, sleepiness results were in line with the hypothesized increase in physiological arousal: both morning and evening subjective sleepiness were reduced more with blue-enriched light exposure compared to the dim light condition as a change from baseline. Although this indicates that light exposure had the expected alerting effect, the lack of association between these changes in sleepiness from the blue-enriched light and dim light condition with changes in HOMA-IR suggests the need to explore additional explanations (other than increased physiological arousal) for the observed changes. Also, morning subjective sleepiness was unexpectedly higher at baseline on the blue-enriched light day (Day 3) compared to the dim light day (Day 2); this phenomenon was consistent among the morning group participants. The reason for this difference is unclear, but we postulate that it may be due to the number of procedures in a short period of time, including the insertion of the intravenous needle for repeated blood sampling on Day 2, which was around the time of the baseline measurement. In addition, there may be an order effect since the dim light day always preceded the blue-enriched light day.

Another possible mechanism implicated in the effects of light exposure on metabolic function is alteration of melatonin levels. While light exposure suppresses melatonin [46] and melatonin downregulates insulin secretion [47, 48], it is unlikely that melatonin plays a role in the changes in insulin observed in the current study. Melatonin production ends, on average, about 2 hours before wake and rises about 14 hours after wake in healthy men and women [49–51]. The acute light exposure in this study (starting 0.5 or 10.5 hours after wake) was given at a time when melatonin levels are typically still low. However, future studies with light exposure over successive days and light exposure at different times of day should determine effects on melatonin phase and its possible contribution to any hunger, weight, or metabolic alterations; there remains a potential role for melatonin in altering hunger, weight regulation [52], and insulin sensitivity [53] in these situations, since light exposure at specific time points can phase shift the circadian clock [54] and day-time light levels influence nocturnal melatonin levels [55].

While the results from the current study support a role for light exposure in regulation of metabolism, the study is not without limitations. For example, we have a small sample that was selected with a limited range of BMI, sleep, and feeding patterns and participants were kept in dim light (< 20 lux) during wake except for the 3 hour light exposure period. It is possible that we would not see the same effects in participants who were obese, had irregular eating and/or sleep patterns, or if participants were in typical room light conditions of 150–500 lux throughout the wake period [56]. Furthermore, food intake was strictly controlled (calories, composition, and timing), which may have also influenced the subjective ratings of food preference since participants knew they could not actively select their preferred food and were required to eat all of their given meals. Future studies with a free choice of the amount and content of meals as well as timing of food intake may allow for elucidation of blue-enriched light exposure’s impacts on behaviors concerning food preference and actual caloric intake. Finally, even though the current study was designed to compare the impact of morning versus evening blue-enriched light exposure, the lack of counterbalancing between the dim and blue-enriched light conditions could be considered a limitation for within group comparisons (dim compared to blue-enriched).

This study highlights the need for further investigation of light exposure in a real-world setting. If the effects of light exposure on glucose metabolism reported in this study are sustained and/or amplified with repeated light exposure and/or under conditions with free access to food, consequences could include changes in hunger, food intake, and/or fat storage, thereby affecting body weight. In addition, if potential changes are dependent on time-of-day, as they seem to be, there may be further consequences since, for example, the timing of food intake has been related to obesity and weight loss success [57, 58]. Also, given that light history influences the magnitude of light exposure’s effects on the circadian system [59], future research is needed to explore whether prior light exposure during the day may impact the effects of evening blue-enriched light exposure on glucose metabolism.

In summary, acute blue-enriched light exposure in the morning and the evening impacts glucose metabolism in healthy adults. Further research is required to determine the mechanism(s) by which this occurs, and how the mechanism(s) may be different at various times of day. Future exploration should also investigate how the relative increase in insulin resistance with light exposure would be beneficial to weight regulation in the morning and detrimental in the evening, as suggested by earlier studies. The data from this study supports the theory that environmental light exposure can impact health and enforces the need for additional research to answer remaining questions to resolve whether manipulating light exposure may be a novel approach for altering glucose metabolism.

## Supporting Information

S1 File Manuscript data. This Excel file contains the data included in this manuscript.
(XLSX)

## References

[1] Ekirch AR. At Day's Close: Night in Times Past. New York: W.W. Norton & Company; 2005.

[2] Wright K.P. Jr., McHill AW, Birks BR, Griffin BR, Rusterholz T, Chinoy ED. Entrainment of the human circadian clock to the natural light-dark cycle. Curr Biol. 2013;23(16):1554–8. 10.1016/j.cub.2013.06.039 23910656 PMC4020279

[3] Piosczyk H, Landmann N, Holz J, Feige B, Riemann D, Nissen C, et al Prolonged sleep under Stone Age conditions. Journal of clinical sleep medicine: JCSM: official publication of the American Academy of Sleep Medicine. 2014;10(7):719–22. 25024647 10.5664/jcsm.3854 PMC4067433

[4] de la Iglesia HO, Fernández-Duque E, Golombek DA, Lanza N, Duffy JF, Czeisler CA, et al Access to Electric Light Is Associated with Shorter Sleep Duration in a Traditionally Hunter-Gatherer Community. J Biol Rhythms. 2015. 10.1177/0748730415590702 PMC5320422 26092820

[5] Stevens RG, Zhu Y. Electric light, particularly at night, disrupts human circadian rhythmicity: is that a problem? Philos Trans R Soc Lond B Biol Sci. 2015;370(1667). 10.1098/rstb.2014.0120 PMC4375361 25780233

[6] Roenneberg T, Kantermann T, Juda M, Vetter C, Allebrandt KV. Light and the human circadian clock. Handbook of experimental pharmacology. 2013;(217):311–31. Epub 2013/04/23. 10.1007/978-3-642-25950-0_13 . 23604485

[7] Cajochen C. Alerting effects of light. Sleep Med Rev. 2007;11(6):453–64. Epub 2007/10/16. S1087-0792(07)00100-1 [pii] 10.1016/j.smrv.2007.07.009 . 17936041

[8] Scheer FA, Morris JS, Shea SA. The internal circadian clock increases hunger and appetite in the evening independent of food intake and other behaviors. Obesity (Silver Spring). 2013;21(3):421–3. 23456944 10.1002/oby.20351 PMC3655529

[9] Van Cauter E, Shapiro ET, Tillil H, Polonsky KS. Circadian modulation of glucose and insulin responses to meals: relationship to cortisol rhythm. Am J Physiol 1992;262(4 pt 1):E467–75. 1566835 10.1152/ajpendo.1992.262.4.E467

[10] Bowen AJ, Reeves RL. Diurnal variation in glucose tolerance. Archives of internal medicine. 1967;119(3):261–4. 6019944

[11] Arble DM, Bass J, Laposky AD, Vitaterna MH, Turek FW. Circadian timing of food intake contributes to weight gain. Obesity (Silver Spring). 2009;17(11):2100–2. Epub 2009/09/05. oby2009264 [pii] 10.1038/oby.2009.264 . 19730426 PMC3499064

[12] Boubekri M, Cheung I.N., Reid K.J., Wang C.H., Zee P.C. Impact of Windows and Daylight Exposure on Overall Health and Sleep Quality of Office Workers—A Case-Control Pilot Study. Journal of Clinical Sleep Medicine. 2014;10(6):603–11. 10.5664/jcsm.3780 24932139 PMC4031400

[13] Gonzalez-Ortiz M, Martinez-Abundis E, Balcazar-Munoz BR, Pascoe-Gonzalez S. Effect of sleep deprivation on insulin sensitivity and cortisol concentration in healthy subjects. Diabetes Nutr Metab. 2000;13(2):80–3. Epub 2000/07/18. . 10898125

[14] Mullington JM, Chan JL, Van Dongen HP, Szuba MP, Samaras J, Price NJ, et al Sleep loss reduces diurnal rhythm amplitude of leptin in healthy men. Journal of neuroendocrinology. 2003;15(9):851–4. . 12899679 10.1046/j.1365-2826.2003.01069.x

[15] Spiegel K, Tasali E, Penev P, Van Cauter E. Brief communication: Sleep curtailment in healthy young men is associated with decreased leptin levels, elevated ghrelin levels, and increased hunger and appetite. Ann Intern Med. 2004;141(11):846–50. Epub 2004/12/08. 141/11/846 [pii]. . 15583226 10.7326/0003-4819-141-11-200412070-00008

[16] Pittendrigh CS. On the mechanism of entrainment of a circadian rhythm by light cycles In: Aschoff J, editor. Circadian Clocks. Amsterdam: North-Holland; 1965 p. 277–97.

[17] Pittendrigh CS. Temporal organization: reflections of a Darwinian clock-watcher. Annu Rev Physiol. 1993;55:16–54. 8466172 10.1146/annurev.ph.55.030193.000313

[18] Brainard GC, Hanifin JP, Greeson JM, Byrne B, Glickman G, Gerner E, et al Action spectrm for melatonin regulation in humans: evidence for a novel circadian photoreceptor. Journal of Neuroscience. 2001;21(16):6405–12. 11487664 10.1523/JNEUROSCI.21-16-06405.2001 PMC6763155

[19] Berson DM, Dunn FA, Takao M. Phototransduction by retinal ganglion cells that set the circadian clock. Science. 2002;295:1070–3. 11834835 10.1126/science.1067262

[20] Lockley SW, Brainard GC, Czeisler CA. High sensitivity of the human circadian melatonin rhythm to resetting by short wavelength light. J Clin Endocrinol Metab. 2003;88(9):4502–5. 12970330 10.1210/jc.2003-030570

[21] Thorne HC, Jones KH, Peters SP, Archer SN, Dijk DJ. Daily and seasonal variation in the spectral composition of light exposure in humans. Chronobiol Int. 2009;26:854–66. 10.1080/07420520903044315 19637047

[22] Wahnschaffe A, Haedel S, Rodenbeck A, Stoll C, Rudolph H, Kozakov R, et al Out of the Lab and into the Bathroom: Evening Short-Term Exposure to Conventional Light Suppresses Melatonin and Increases Alertness Perception. International journal of molecular sciences. 2013;14(2):2573–89. 10.3390/ijms14022573 23358248 PMC3588003

[23] Jung CM, Khalsa SB, Scheer FA, Cajochen C, Lockley SW, Czeisler CA, et al Acute effects of bright light exposure on cortisol levels. J Biol Rhythms. 2010;25(3):208–16. Epub 2010/05/21. 25/3/208 [pii] 10.1177/0748730410368413 . 20484692 PMC3686562

[24] Ishida A, Mutoh T, Ueyama T, Bando H, Masubuchi S, Nakahara D, et al Light activates the adrenal gland: timing of gene expression and glucocorticoid release. Cell Metab. 2005;2(5):297–307. Epub 2005/11/08. S1550-4131(05)00269-X [pii] 10.1016/j.cmet.2005.09.009 . 16271530

[25] Rizza RA, Mandarino LJ, Gerich JE. Cortisol-induced insulin resistance in man: impaired suppression of glucose production and stimulation of glucose utilization due to a postreceptor detect of insulin action. J Clin Endocrinol Metab. 1982;54(1):131–8. Epub 1982/01/01. . 7033265 10.1210/jcem-54-1-131

[26] Perry CG, Spiers A, Cleland SJ, Lowe GD, Petrie JR, Connell JM. Glucocorticoids and insulin sensitivity: dissociation of insulin's metabolic and vascular actions. J Clin Endocrinol Metab. 2003;88(12):6008–14. Epub 2003/12/13. . 14671204 10.1210/jc.2002-021605

[27] Danilenko KV, Mustafina SV, Pechenkina EA. Bright light for weight loss: results of a controlled crossover trial. Obes Facts. 2013;6(1):28–38. 10.1159/000348549 . 23429094 PMC5644670

[28] Figueiro MG, Plitnick B, Rea MS. Light modulates leptin and ghrelin in sleep-restricted adults. Int J Endocrinol. 2012;2012:530726 10.1155/2012/530726 22988459 PMC3440859

[29] Dunai A, Novak M, Chung SA, Kayumov L, Keszei A, Levitan R, et al Moderate exercise and bright light treatment in overweight and obese individuals. Obesity (Silver Spring). 2007;15(7):1749–57. 10.1038/oby.2007.208 . 17636093

[30] Reid KJ, Santostasi G, Baron KG, Wilson J, Kang J, Zee PC. Timing and intesity of light correlate with body weight in adults. PLoS One. 2014;9(4):e92251 10.1371/journal.pone.0092251 24694994 PMC3973603

[31] van Amelsvoort LG, Schouten EG, Kok FJ. Duration of shiftwork related to body mass index and waist to hip ratio. Int J Obes Relat Metab Disord. 1999;23:973–8. 10490804 10.1038/sj.ijo.0801028

[32] Schernhammer ES, Thompson CA. Light at night and health: the perils of rotating shift work. Occupational and environmental medicine. 2011;68(5):310–1. 10.1136/oem.2010.058222 20921271 PMC8931862

[33] Fonken LK, Workman JL, Walton JC, Weil ZM, Morris JS, Haim A, et al Light at night increases body mass by shifting the time of food intake. Proc Natl Acad Sci U S A. 2010;107(43):18664–9. Epub 2010/10/13. 1008734107 [pii] 10.1073/pnas.1008734107 20937863 PMC2972983

[34] Coomans CP, van den Berg SA, Houben T, van Klinken JB, van den Berg R, Pronk AC, et al Detrimental effects of constant light exposure and high-fat diet on circadian energy metabolism and insulin sensitivity. FASEB journal: official publication of the Federation of American Societies for Experimental Biology. 2013;27(4):1721–32. 10.1096/fj.12-210898 . 23303208

[35] Beck AT, Steer RA. Internal consistencies of the original and revised Beck Depression Inventory. Journal of clinical psychology. 1984;40(6):1365–7. . 6511949 10.1002/1097-4679(198411)40:6<1365::aid-jclp2270400615>3.0.co;2-d

[36] Harris JA, Benedict FG. A biometric study of human basal metabolism. Proceedings of the National Academy of Sciences. 1918;4(12):370–3. 10.1073/pnas.4.12.370 PMC1091498 16576330

[37] Stevens SS. A metric for the social consensus. Science. 1966;151(710):530–41. Epub 1966/02/04. . 5323509 10.1126/science.151.3710.530

[38] Spiegel K, Leproult R, L'Hermite-Baleriaux M, Copinschi G, Penev PD, Van Cauter E. Leptin levels are dependent on sleep duration: relationships with sympathovagal balance, carbohydrate regulation, cortisol, and thyrotropin. J Clin Endocrinol Metab. 2004;89(11):5762–71. Epub 2004/11/09. 89/11/5762 [pii] 10.1210/jc.2004-1003 . 15531540

[39] Spiegel K, Knutson K, Leproult R, Tasali E, Van Cauter E. Sleep loss: a novel risk factor for insulin resistance and Type 2 diabetes. J Appl Physiol. 2005;99(5):2008–19. Epub 2005/10/18. 99/5/2008 [pii] 10.1152/japplphysiol.00660.2005 . 16227462

[40] Basu A, Betts NM, Leyva MJ, Fu D, Aston CE, Lyons TJ. Acute cocoa supplementation increases postprandial HDL cholesterol and insulin in obese adults with type 2 diabetes after consumption of a high-fat breakfast. J Nutr. 2015;145(10):2325–32. 10.3945/jn.115.215772 26338890 PMC4580960

[41] Hancox RJ, Landhuis CE. Correlation between measures of insulin resistance in fasting and non-fasting blood. Diabetol Metab Syndr. 2011;3:23 10.1186/1758-5996-3-23 21899745 PMC3177770

[42] Akerstedt T, Gillberg M. Subjective and objective sleepiness in the active individual. Int J Neurosci. 1990;52(1–2):29–37. Epub 1990/05/01. . 2265922 10.3109/00207459008994241

[43] Kaida K, Takahashi M, Akerstedt T, Nakata A, Otsuka Y, Haratani T, et al Validation of the Karolinska sleepiness scale against performance and EEG variables. Clin Neurophysiol. 2006;117(7):1574–81. Epub 2006/05/09. S1388-2457(06)00142-8 [pii] 10.1016/j.clinph.2006.03.011 . 16679057

[44] Klingenberg L, Chaput JP, Holmback U, Visby T, Jennum P, Nikolic M, et al Acute Sleep Restriction Reduces Insulin Sensitivity in Adolescent Boys. Sleep. 2013;36(7):1085–90. 10.5665/sleep.2816 23814346 PMC3669075

[45] Kostoglou-Athanassiou I, Treacher DF, Wheeler MJ, Forsling ML. Bright light exposure and pituitary hormone secretion. Clin Endocrinol (Oxf). 1998;48(1):73–9. Epub 1998/03/24. . 9509071 10.1046/j.1365-2265.1998.00355.x

[46] Lewy AJ, Wehr TA, Goodwin FK, Newsome DA, Markey SP. Light suppresses melatonin secretion in humans. Science. 1980;210(1267–1269). 10.1126/science.7434030 7434030

[47] Boden G, Ruiz J, Urbain JL, Chen X. Evidence for a circadian rhythm of insulin secretion. American Journal of Physiology. 1996;271(2):E246–52. 8770017 10.1152/ajpendo.1996.271.2.E246

[48] Peschke E, Bahr I, Muhlbauer E. Melatonin and Pancreatic Islets: Interrelationships between Melatonin, Insulin and Glucagon. Int J Mol Sci. 2013;14(4):6981–7015. 10.3390/ijms14046981 23535335 PMC3645673

[49] Tzischinsky O, Shlitner A, Lavie P. The Association between the Nocturnal Sleep Gate and Nocturnal Onset of Urinary 6-Sulfatoxymelatonin. Journal of Biological Rhythms. 1993;8(3):199–209. 8280909 10.1177/074873049300800303

[50] Burgess HJ, Fogg LF. Individual Differences in the Amount and Timing of Salivary Melatonin Secretion. PLoS ONE 2008;3(8):e3055 10.1371/journal.pone.0003055 18725972 PMC2516604

[51] Benloucif S, Guico MJ, Reid KJ, Wolfe LF, L'Hermite-Baleriaux M, Zee PC. Stability of melatonin and temperature as circadian phase markers and their relation to sleep times in humans. J Biol Rhythms. 2005;20(2):178–88. . 15834114 10.1177/0748730404273983

[52] Puchalski SS, Green JN, Rasmussen DD. Melatonin effect on rat body weight regulation in response to high-fat diet at middle age. Endocrine. 2003;21:163–7. 12897381 10.1385/ENDO:21:2:163

[53] McMullan C, GC C, Schernhammer E, Forman J. Association of nocturnal melatonin secretion with insulin resistance in nondiabetic young women. Am J Epidemiol. 2013;178(2):231–8. 10.1093/aje/kws470 23813704 PMC3937598

[54] Khalsa SB, Jewett ME, Cajochen C, Czeisler CA. A phase response curve to single bright light pulses in human subjects. J Physiol Anthropol Appl Human Sci. 2003;549(945–952). 10.1113/jphysiol.2003.040477 PMC2342968 12717008

[55] Obayashi K, Saeki K., Iwamoto J., Okamoto N., Tomioka K., Nezu S., Ikada Y., Kurumatani N. Positive effect of daylight exposure on nocturnal urinary melatonin excretion in the elderly: a cross-sectional analysis of the HEIJO-KYO study. J Clin Endocrinol Metab. 2012;97:4166–73. 10.1210/jc.2012-1873 22948764

[56] Osterhaus W. Office lighting: a review of 80 years of standards and recommendations. Toronto, Ontario, Canada1993.

[57] Garaulet M, Gomez-Abellan P, Alburquerque-Bejar JJ, Lee YC, Ordovas JM, Scheer FA. Timing of food intake predicts weight loss effectiveness. International journal of obesity. 2013;37:604–11. 10.1038/ijo.2012.229 23357955 PMC3756673

[58] Garaulet M, Gomez-Abellan P. Timing of food intake and obesity: A novel association Physiol Behav. 2014;134:44–50. 10.1016/j.physbeh.2014.01.001 24467926

[59] Chang AM, Scheer FA, Czeisler CA, Aeschbach D. Direct effects of light on alertness, vigilance, and the waking electroencephalogram in humans depend on prior light history. Sleep. 2013;36(8):1239–46. 10.5665/sleep.2894 23904684 PMC3700721

